# Sexual Risk Behaviours and Sexual Abuse in Persons with Severe Mental Illness in Uganda: A Qualitative Study

**DOI:** 10.1371/journal.pone.0029748

**Published:** 2012-01-09

**Authors:** Patric Lundberg, Eva Johansson, Elialilia Okello, Peter Allebeck, Anna Thorson

**Affiliations:** 1 Division of Social Medicine, Department of Public Health Sciences, Karolinska Institute, Stockholm, Sweden; 2 Division of Global Health/IHCAR, Department of Public Health Sciences, Karolinska Institute, Stockholm, Sweden; 3 Nordic School of Public Health, Gothenburg, Sweden; 4 Department of Psychiatry, Makerere University, Kampala, Uganda; University of Cape Town, South Africa

## Abstract

Persons with severe mental illness (SMI) engage in risky sexual behaviours and have high prevalence of HIV in high-income countries. Little is known about sexual behaviours and HIV risk among persons with SMI in sub-Saharan Africa. In this qualitative study we explored how SMI may influence sexual risk behaviours and sexual health risks in Uganda. Individual semi-structured interviews were conducted with 7 male and 13 female psychiatric patients aged 18–49 years. Participants were interviewed in hospital when clinically stable and capable of giving informed consent. Interview transcripts were analysed using manifest content analysis, generating the categories: (1) casual sex during illness episodes, (2) rape by non-partners, (3) exploitation by partners, (4) non-monogamous partners, and (5) sexual inactivity. Our findings suggest that SMI exacerbated sexual vulnerability in the women interviewed, by contributing to casual sex, to exploitative and non-monogamous sexual relationships, and to sexual assault by non-partners. No link could be established between SMI and increased sexual risk behaviours in the men interviewed, due to a small sample of men, and given that men's accounts showed little variability. Our findings also suggest that SMI caused sexual inactivity due to decreased sexual desire, and in men, due to difficulties forming an intimate relationship. Overall, our study highlights how SMI and gender inequality can contribute to the shaping of sexual risk behaviours and sexual health risks, including HIV risk, among persons with SMI in this Ugandan setting.

## Introduction

Improving health and rights of persons with mental illness is a global public health priority [1]. Socio-economic disadvantage, stigmatisation and human rights violations make persons with mental illness vulnerable beyond the effects of the mental illness itself [2]. One domain of vulnerability concerns sexual health and HIV risk.

In high-income countries, persons with severe mental illness (SMI; i.e. schizophrenia, bipolar affective disorder and other psychoses) have been found to have a higher HIV prevalence than the general population [3], attributed to sexual risk behaviours and injecting drug use [4]. High-risk behaviours among persons with SMI have been suggested to stem from both psychiatric symptoms and from the social consequences of the illness (e.g. homelessness, substance use) [4], [5]. In addition, childhood sexual abuse may be a risk factor for sexual risk behaviours in persons with SMI [6].

In low- and middle-income countries, lack of psychiatric services and widespread mental illness stigma are structural factors that exacerbate the social vulnerability of persons with SMI [7], [8] hypothetically also increasing their vulnerability to HIV infection [9]. Many low- and middle-income countries in sub-Saharan Africa have high, or very high, HIV prevalence. The extent to which persons with SMI are disproportionally vulnerable to HIV in these countries therefore has public health relevance.

Studies on HIV prevalence among persons with SMI in sub-Saharan African low- and middle income settings have reported HIV prevalence estimates generally similar to or higher than those in the respective general populations [10], [11], [12], [13], suggesting that persons with SMI may indeed be at high risk of HIV infection. However, the studies on sexual risk behaviours among persons with SMI in sub-Saharan Africa are few [13], [14], [15], [16] and the understanding of how sexual health risks are shaped in this group is limited. Thus, there is a public health need to explore sexual behaviours and risks among persons with SMI in sub-Saharan African low- and middle-income countries in order to inform future interventions targeting this group.

Qualitative methodology may enhance the understanding of quantitative data, and is useful when the area of investigation is unexplored. Qualitative studies have provided important information on sexual behaviours and experiences among persons with SMI in the United States [17], [18], [19], [20], in Brazil [21], [22] and in India [23]. However, to our knowledge, no qualitative study has explored sexual risk behaviours and sexual health risks as described by the persons with SMI themselves in a sub-Saharan African context.

Thus, we conducted semi-structured interviews with persons with SMI in Uganda in order to understand how having a SMI may influence sexual risk behaviours and sexual health risks in a low-income sub-Saharan African country with high HIV prevalence.

## Methods

### Study setting

The study was performed in Butabika and Mulago Hospitals in the Ugandan capital Kampala. HIV prevalence among first-time psychiatric admissions in Butabika Hospital in 2005 was 18.4% [13]. The national HIV prevalence among adults at the time of that study was 6.4% [24].

Butabika and Mulago are both national referral facilities, with patients originating from all over Uganda. Butabika is situated in the outskirts of Kampala and has about four hundred fifty beds for psychiatric inpatients, although on most occasions about six to seven hundred patients are admitted. The hospital also offers outpatient psychiatric services. Mulago is situated in the city centre and has about fifty beds for psychiatric inpatients, and also offers outpatient psychiatric services. Patients are generally brought to the hospitals for admission by relatives or by the police. Men and women have separate wards in both hospitals. The majority of patients are discharged after approximately two or three weeks (Dr Noeline Nakasujja, personal communication, May 2011).

### Participants

Twenty participants were individually interviewed using a semi-structured interview design. We *a priori* deemed a sample size of 15 to 20 participants to be sufficient in order to capture a range of personal experiences of both men and women. During the course of data collection the decision was taken to include 20 participants, given that some of the completed interviews were not rich in content and thus would contribute little information to the study aim.

Inclusion criteria were: age 18–49 years, diagnosis of schizophrenia, bipolar affective disorder or depression, and mental capacity to give informed consent. Capacity to give informed consent was assessed both by the referring clinician and by the interviewer based on: (1) capacity to understand the information given about the study and to express a choice based on this information, (2) insight about having a mental health problem, and (3) capacity to participate in a focussed discussion. At the time of the interview, all inpatients had been cleared for discharge. Outpatients were interviewed after their consultation with the clinician.

Participants were recruited from psychiatric outpatients departments and from convalescent wards by means of purposive sampling. We selected participants with different gender, diagnosis and in- or outpatient status, in order to obtain variability with respect to sexual risk behaviours and sexual health risks. After the initial interviews we noted that less variability was found in men's accounts than in women's accounts, and we therefore decided to interview a greater proportion of women than of men. Similarly, little variability was found among persons with depression and we therefore decided to include relatively fewer persons with depression than with other diagnoses. Towards the end of the data collection we decided to purposively include men and women with SMI who were HIV positive. However, while four HIV positive female patients were thus included in the study we failed to identify any eligible HIV positive male patient within the remaining data collection period.

Interviews were successively planned and scheduled during the course of the data collection. On interviewing days, the interviewer visited the relevant ward or outpatient department and informed the attending clinician about the type of participant (i.e. gender, diagnosis) desired. Clinicians then purposively selected potential participants from the patients attending their services. The average number of patients admitted in each convalescent ward during the study period was estimated at 100 to 130. The number of patients attending each outpatients' department during the study period was estimated at 60 to 120 per day.

In total, 26 persons with SMI were thus selected by clinicians and referred to the interviewers. After re-assessment of the mental state by the interviewers, 22 persons were found to be capable of giving informed consent, and of these 20 consented to participate in the study. Two participants refused to participate due to lack of time.

### Data collection

Data was collected from November 2009 until January 2010. Four Ugandan clinical psychologists who were not known by the study participants conducted the interviews in Luganda or English, depending on the participant's preference. Interviewers and interviewees were sex-matched. Interviewers were trained by the first and third authors (PL and EO). Training focussed on ethical and technical issues pertinent to interviewing persons with SMI about sensitive topics, and included mock interviews followed by feedback.

A semi-structured interview guide with open-ended questions was used covering the following topics: current living situation, perceived influence of mental illness on life, perceptions and attitudes towards sex, experiences of sexual relationships, experiences of sexual abuse, knowledge and attitudes towards HIV, and perceived influence of mental illness on sexual life. A summary of the main questions from the interview guide are presented in Table 1, although it should be noted that minor modifications to the interview guide were made throughout the field-work: For instance, explicit questions about sexual abuse were added to the interview guide when the first rounds of interviews had been completed. Moreover, depending on the flow and content of conversation interviewers probed for more information, added new questions, skipped questions or reversed the order of questions. Socio-demographic information and information about HIV status were collected at the end of the interview using structured questions. Psychiatric diagnoses were extracted from patient charts and had been made by the treating psychiatrist at transferral from the acute ward, or at the current visit at the outpatient department.

**Table 1:** Main questions from interview guide.

**1. Living situation.**
Please tell me about:
- your living situation (probe: residence, family, income etc).
- your relationship with your family.
- your relationship with people outside your family.
**2. Mental illness.**
Please tell me about:
- the last time you fell ill.
- how your life has changed since the first time you fell ill.
- how your life changes every time your illness gets worse.
**3. Sex.**
Please tell me about:
- what you think sex means to people in general.
- what sex means in your life.
- the last person that you had sex with.
- other persons that you have had sex with.
- whether you have ever been forced to have sex.
- whether you have ever forced somebody to have sex.
**4. HIV.**
Please tell me about:
- whether you think there is a link between sex and HIV. What link?
- whether you have ever had an HIV test. What result?
- whether the test result has influenced your sexual life. How?
- whether you do anything to protect yourself against HIV? What?
- whether you do anything to protect your sex partners against HIV? What?
**5. Mental illness and sex.**
Please tell me about:
- whether having a mental problem may influence one's sexual life. How?
- whether your mental problem has influenced your sexual life? How?

Interviews were conducted in secluded rooms inside the hospital premises. Interviewers wore civil clothes and explained their independence from the hospital staff. Interview length ranged from about 30 to 90 minutes. Interviews were audio recorded. Interviewers listened to audio recordings, translated and transcribed verbatim into English.

PL attended each interviewer's first interview in order to supervise the informed consent procedure, to provide feedback on interviewing techniques and to observe participants' reactions to the questions asked. After each subsequent interview, discussions were held with the interviewer, and interview content and quality was discussed in order to identify needs to revise the interview guide.

### Ethics statement

All study procedures were approved by the Makerere University Research and Ethics Committee and by the Uganda National Council of Science and Technology. The consent form was read to all participants, and all participants gave written or audio recorded oral consent. Oral consent was accepted given the high illiteracy rate in the study population.

Participants were informed that their participation in the study and its results would not influence their treatment. Confidentiality was guaranteed with no information given from the research assistants to any person outside the research team, including to the staff in the psychiatric wards. Psychosocial and/or HIV counselling was provided by interviewers after the interview when indicated. Participants received 5000 Ugandan Shillings (2.5 US dollars) as compensation for their time.

### Data analysis

PL and EO read interview transcripts during the data collection in order to guide the purposive sampling. Interview transcripts were compared to the original audio recordings when indicated, in order to assess the quality of the transcription procedure.

After the data collection, interview transcripts were read several times in order to get a deeper understanding of the interview material in its entirety. A case-summary for each participant was also developed in order for the analysts to become familiarised with that person's narrative. Subsequently, PL analysed interview transcripts using manifest content analysis [25]. Manifest content analysis was deemed appropriate since the aim was to understand how SMI might influence sexual risk behaviours and sexual health risks, based on the face-value of participants' accounts.

First, meaning units (e.g. sentences) representing or deemed relevant to sexual risk behaviours and sexual health risks were labelled using descriptive codes. Second, the list of codes was examined and identical codes were merged. Third, codes were aggregated into categories based on similarities and differences. The names of these categories were inductively derived from the data. The NVIVO 9 software was used in order to label text fragments with codes, to arrange the codes into categories and to retrieve the original text from the codes and categories. In order to achieve analyst triangulation, all authors read a selection of transcripts, and PL, EJ and AT discussed the extent to which categories reflected the data, and reached consensus on the final list of five categories.

The findings were discussed with two independent Ugandan psychiatrists and one Ugandan public health specialist in order to enhance the credibility of the findings and ensure that the interpretations made were relevant to the study context.

## Results

Participants' socio-demographic and clinical characteristics are presented in Table 2.

**Table 2:** Socio-demographic and clinical characteristics of participants.

Characteristic		Men (n = 7)	Women (n = 13)	Total (n = 20)
		n (%)	n (%)	n (%)
Diagnosis	Bipolar affective disorder	4 (57)	6 (46)	10 (50)
	Schizophrenia	2 (29)	4 (31)	6 (30)
	Depression	1 (14)	3 (23)	4 (20)
Type of patient	Inpatient	4 (57)	9 (69)	13 (65)
	Outpatient	3 (43)	4 (31)	7 (35)
Age	18–29	4 (57)	7 (54)	11(55)
	30–39	1 (14)	4 (31)	5 (25)
	40–49	2 (29)	2 (15)	4 (20)
Place of residence	Kampala district	4 (57)	5 (38)	9(45)
	Other district	3 (43)	8 (62)	11(55)
Education^1^	Primary school (≤7 years)	0 (0)	6 (50)	6 (35)
	Secondary school (>8 years)	5 (100)	6 (50)	11 (65)
Occupation^2^	Unemployed	2 (29)	10 (83)	12 (63)
	Informal employment	4 (57)	2 (17)	6 (32)
	Formal employment	1 (14)	0 (0)	1(5)
Marital status	Never married	7 (100)	5 (39)	12 (60)
	Married	0 (0)	2 (15)	2 (10)
	Divorced/Separated/Widowed	0 (0)	6 (46)	6 (30)
HIV status	Negative or unknown	7 (100)	9 (69)	16 (80)
	Positive	0 (0)	4 (31)	4 (20)

1 Information was missing for two men and one woman.

2 Information was missing for one woman.

The analysis of the interview transcripts suggested that for many study participants the SMI had constituted an important influence on sexual behaviours and sexual health risks. The manifest content analysis of the transcripts generated the following five categories: (1) casual sex during illness episodes, (2) rape by non-partners, (3) exploitation by partners, (4) non-monogamous partners, and (5) sexual inactivity. Table 3 provides an overview of categories and their underlying descriptive codes.

**Table 3:** Overview of the study findings.

Descriptive code^1^	Category
Sex during episodes	Casual sex during illness episodes
Sex because wanted to die	
Wanting immediate satisfaction	
Raped outside home by stranger	Rape by non-partners
Raped at home by intruder	
Sex for money	Exploitation by partners
Sex for psychiatric treatment	
Reactions to partner unfaithfulness	Non-monogamous partners
Absence from home creates opportunity	
Illness made partner get other partner	
Lack of desire and sexual dysfunction	Sexual inactivity
Perceived unattractiveness in men	
Money needed to get partner	

1 Descriptive codes frequently refer to meaning units (i.e. text fragments) from more than one study participant.

The range of sexual experiences was wider among the thirteen women interviewed than among the seven men. Moreover, most of the men interviewed reported little or no past and current sexual activity. Thus, more information was obtained regarding sexual risk behaviours and sexual health risks among women than among men. Consequently, women's accounts contributed data to all five content categories, while men's accounts contributed data to only two categories: ‘non-monogamous partners’ and ‘sexual inactivity’.

Four HIV positive women, but no HIV positive man, were included as study participants, and the accounts of these women were rich in examples of how SMI may influence sexual risk behaviours and sexual health risks. The stories of these women contributed data to three of the five content categories: ‘casual sex during illness episodes’, ‘rape by non-partners’, and ‘non-monogamous partners’. However, no category was based solely on data from HIV positive persons.

Categories are described below, together with illustrative citation examples.

### Casual sex during illness episodes

One man and several women reported unprotected sex with casual partners after the onset of the mental illness. Some participants' stories suggested that illness symptoms could potentially have increased participants' sexual risk-taking. Three female participants with bipolar disorder described periods of increased sexual activity coinciding with probable illness episodes. For instance, one HIV infected woman with bipolar disorder described how when mentally distressed she had sex with several casual partners because ‘she wanted to die’, suggesting that casual sex was a self-destructive behaviour for this woman:

“This year… this year is when I did all those things [had casual sex] because I was fed up with the world and I wanted to die. // I even set the house on fire when I was inside so that I would die, but I did not die. I felt…I was fed up with the world. // I was miserable…

*But would you get the people to have sex with?*

Yes! I would get them when I would go to the club. You can't fail to get a man who approaches you when you go to a club. So I get them and we go to the lodge [a hotel]. That is where we sleep.”

(ID 14, woman, 30–39 years, bipolar affective disorder, HIV positive)

The story of another female participant with bipolar disorder suggested that during illness episodes she may be more likely to make impulsive decisions about sex. This participant answers below a hypothetical question about what she would do if a man approached her and proposed her to have sex during an illness episode:

*“How about if you are sick, what do you think you would say?*

There… hmm… there I have nothing to do, because the mind is not thinking at that point, because then you are thinking about those very minutes, like they say these days: *kagwiirawo* [immediate satisfaction].

(ID 10, woman, 18–29 years, bipolar affective disorder, HIV negative)

### Rape by non-partners

Five female participants described experiences of rape by non-partners after the first onset of their illness. At least three of these women were raped during illness episodes. One woman described how she was chased away from her sister's home following a conflict during a probable manic episode. The woman, who had already been sexually assaulted during an earlier illness episode, spent several nights on the streets of Kampala, resulting in a second rape by a stranger:

“// For me, when I slept on the street I was raped again. // I reached a point where these street men stole all my clothes. // When night came one of them took advantage of me. He was a boda-boda man [motorcycle taxi rider].”

(ID 10, woman, 18–29 years, bipolar affective disorder, HIV negative)

Another woman reported being raped by a person who was doing construction work in the house where she was temporarily staying when ill.

Other women described being sexually assaulted seemingly outside of illness episodes. One woman with schizophrenia described being raped at home by an unknown intruder, and another woman with schizophrenia described how she was raped by a motorcycle taxi rider when she refused to have sex with him:

“He found me on the way…he broke my back.

*Where did it happen?*

I had come from the other side, and come to Kampala… and he broke my back.

*Do you remember what happened before that?*

No… He just pushed me down suddenly.

*Did you know that man?*

I didn't know him.”

(ID 16, woman, 18–29 years, schizophrenia, HIV positive)

### Exploitation by partners

Some women's stories described how partners took advantage of them when in situations of dependence. One woman with bipolar disorder described how when her symptoms came back her mother beat her and forced her to go to her grandparents' place in another part of Kampala, without supplying her with any money. The participant engaged in several sexual relationships in the new environment partly in order to obtain economic support, and described partners taking advantage of her economic dependence:

“// They [the sexual partners] promise you money sometimes but do not give it to you… some give you, some don't… Well, these two gave me money and to be honest I used it”

(ID 11, woman, 18–29 years, bipolar affective disorder, HIV negative)

One woman was sexually exploited by the clinician treating her mental illness, who turned a friendly relationship into a sexual relationship when the participant's family was no longer able, or willing, to pay for the expensive psychiatric medication. Although the woman initially interpreted the relationship as romantic, she later felt unable to leave the relationship since no one at home would provide the medication. The woman described how her family had given her away to the clinician:

“Sometimes our parents are the ones who put us in such temptations. Because when the man said that he was going to treat me, they should have stopped him and said NO [tapping table], we are going to give you money. But they said thank you *musawo* [clinician]. Now, doesn't that mean that you have fully given away your daughter? Yes, you have given away your daughter. Do you think that that man can treat me without anything?”

(ID 8, woman, 18–29 years, bipolar affective disorder, HIV negative)

The economic dependence of this female participant in relationship to her abusive partner made her powerless in decision-making about sex and condom use.

### Non-monogamous partners

Several women described intimate partners who had concurrent sexual partners, either official co-wives or unofficial extramarital partners. For many women this was a source of constant worry and distress. Some women who were able to obtain economic support from other sources chose to leave the non-monogamous partner and go back to their family and relatives.

Similarly, the only man who reported having had an unfaithful intimate partner described how he had left his partner when he learnt about her unfaithfulness. However, most women remained in the non-monogamous relationship while expressing varying degrees of questioning and acceptance for their partner's behaviour:

“Of course that is what men do [have extramarital partners]. Now, you see for me I am in the village, I cannot start putting limitations on him that he should only sleep with me. You know the nature of men…”

(ID 17, woman, 30–39 years, depression, HIV negative)

Some female participants described how their SMI indirectly contributed to their partner's unfaithfulness. One woman described how her absence from home during illness episodes made her partner have sex with another woman.

“// When I am away [in the hospital], my husband has got another partner and he is going to have sex with that one and then finally contaminates me with the disease [HIV].”

(ID 6, woman, 18–29 years, depression, HIV negative)

Another female participant's story suggests that the woman's aggressive behaviour during illness episodes might have contributed to her husband's unfaithfulness, and eventually, to his wish for separation.

“My husband…When I gave birth to my first child in 1990, and I got the mental illness, we went to the witchdoctors and I got fine. He took me back home. When I got the second born the mental illness came back. By then he had got another woman. // He went and told my mother: ‘I have failed with your daughter, and because of that you come for her property. // She is very bad-tempered and she can even kill me. I now have another wife that I can agree with’. They packed my things, he did not steal them. We packed them and took them back to our home.”

(ID 13, woman, 30–39 years, bipolar affective disorder, HIV positive)

One participant described how her husband's mother put pressure on the husband to look for another partner and terminate the relationship with the mentally ill woman. However, the husband resisted and stayed in the relationship.

### Sexual inactivity

Several men and women described how their mental illness contributed to sexual inactivity. Some participants became irritable and aggressive with no desire for intimacy when symptoms worsened. Other participants described how they withdrew from all kinds of social interaction when ill. One male participant diagnosed with depression described how ‘weakness’ had made him loose interest in sex:

“This sickness made me loose interest in having sex. I don't have the energy to do it because I'm weak and you need to be fit to have sex. That's the biggest challenge I have got as a result of my sickness. So I'm basically not into issues of sex because of that. Possibly it has helped me to protect myself [against HIV] that way.”

(ID 5, man, 18–29 years, depression, HIV negative)

Most men, but no woman, described with considerable frustration how being seen as ‘mad’ or ‘mental’ makes it difficult to attract potential partners. One man described how if a woman has seen him ‘when moving on the road’ she will never consider him eligible as a potential partner. Another man felt that other persons knew that his future was spoilt due to his mental illness and that women therefore did not show him any interest:

“… since I became sick, there is no hope [in me]. So, what do women do? They follow men of hope. They can't follow men of no hope.”

(ID 19, man, 18–29 years, bipolar affective disorder, HIV negative)

In addition, male participants described how the mental illness leads to loss of income and poor economy. Several men were no longer able to work as before the illness started. One man described how poor economy devaluates the mentally ill men in the eyes of potential partners and leads to sexual isolation:

“Surely for me at my age I would desire to get children, but ever since I got this illness my life has changed. You know, ladies cannot come to you after knowing that you're a mental patient. That's why I have spent seven years without having sex. // You know, women love men who have money. Now, like me, how do I go to church requesting to marry someone when I don't even have enough money to support myself? It is so hard.”

(ID 3, man, 40–49 years, schizophrenia, HIV negative)

Also one woman felt that men showed her less interest as a potential partner after she lost her job. The woman suggested that men prefer women who have a job because such women less frequently ask for money and gifts.

## Discussion

Our findings suggest that SMI exacerbated sexual vulnerability in the women interviewed, by contributing to casual sex, to exploitative and non-monogamous sexual relationships, and to sexual assault by non-partners. No clear link could however be established between SMI and increased sexual risk behaviours or sexual health risks in the men interviewed, due to a small sample of men and given that men's accounts showed little variability. Our findings also indicate that SMI caused sexual inactivity due to decreased sexual desire, and in men, due to difficulties forming an intimate relationship. Overall, our study highlights how SMI and gender inequality can contribute to the shaping of sexual risk behaviours and sexual health risks, among persons with SMI in this Ugandan setting.

The observed relationship between SMI and sexual health risks in the women in our study could hypothetically translate into an increased HIV incidence in this group. Women have higher prevalence of HIV infection than men in sub-Saharan Africa in general [26]. The only published HIV prevalence study among persons with SMI in Uganda reported that gender differences in HIV prevalence were more marked among persons admitted for SMI (women: 30.2%, men: 9.6%) [13] than in the general population (women: 7.5%, men: 5.0) [24].

Our findings indicate that uncontrolled psychiatric symptoms may potentially contribute to casual sexual encounters. Links between psychiatric symptoms and risky sexual behaviours were found in this study, and have previously been described [27]. The possibility of a causal pathway from uncontrolled psychiatric symptoms to sexual risk-taking warrant concern in a low-income country like Uganda, where most persons with SMI do not have access to treatment [28]. In addition, HIV prevalence among persons with SMI in Uganda is high [13], and HIV infected persons with untreated psychiatric illness could thus hypothetically contribute to further transmission of HIV. Indeed, the current study provides examples of sexual risk behaviours among persons with SMI who are HIV infected.

Several of the women we interviewed described being sexually assaulted by strangers. Sexual violence against women is common in Uganda, but in general the perpetrator is known by the victim [29]. Studies in high-income countries suggest that women with SMI are at increased risk of sexual violence both within and outside intimate relationships [30], [31], [32]. To our knowledge, this study is the first to describe sexual violence against women with SMI in a sub-Saharan African context. Victimisation theories have been used to explain why persons with SMI seem to be at increased risk of violence victimisation globally [33]. According to the ‘Routine Activities Theory’ [34], victimisation results from the co-occurrence of likely offenders, suitable targets, and the absence of capable guardians. Applying this theory to the Ugandan context several factors could hypothetically contribute to sexual violence against women with SMI. Firstly, sexual violence is common within intimate relationships [35], [36], [37], arguably lowering many men's psychological threshold for perpetrating sexual violence in general. Secondly, access to mental health treatment is limited, and women with inadequately treated psychiatric symptoms could constitute ‘suitable targets’. Thirdly, stigmatising attitudes against persons with SMI are widespread [38], potentially also among those supposed to provide protection (e.g. family members, policemen). Indeed, during an informal conversation with a female patient who was not a study participant, this woman described being raped by a security guard inside the psychiatric ward (data not shown). Five of the female participants reported being raped by non-partners, and our data raise questions about the extent of this problem in Uganda.

Some female participants described being sexually exploited due to economic and emotional dependence by persons who were, or wanted to become, their intimate partners. Economic dependence on intimate partners has been suggested to contribute to Ugandan women's low negotiating power in decision-making about sex [39]. Our findings suggest that SMI exacerbated these Ugandan women's economic dependence on men, due to loss of income, treatment related costs and poor social support from relatives. For instance, women described family members refusing to provide the economic support women needed to buy the psychiatric medication. In addition, some women's stories suggested that family members deliberately distanced themselves from, or rejected, the mentally ill participant. Family members of persons with SMI in resource-poor settings face economic burdens [40] and the risk of family stigma [41]. Withdrawal of economic support from the mentally ill person might result from economic hardship, but could also provide a strategy to avoid stigma contamination. Economic dependence put some women in situations where sex had to be traded for resources, and where women had little control over when to have sex, and whether to use a condom, seemingly increasing these women's HIV risk.

Many of the women interviewed had non-monogamous partners and unstable intimate relationships. Extramarital sex among men may be prevalent in Uganda in general [29], [42], [43], and our data raise the question as to whether women with SMI might be particularly exposed to non-monogamous intimate partners. Lack of alternative sources of support made some women remain in unequal relationships with non-monogamous partners. In addition, several women had been repeatedly admitted to hospital, and one female participant explained that frequent hospitalisations create opportunities for extramarital sex for the male partner staying behind. Absence from home has indeed been found to be associated with sexual risk-taking in partners staying at home, in a sub-Saharan African setting [44]. Having sexual partners who have concurrent partners may constitute a key risk factor for HIV in sub-Saharan Africa [45], although this has been questioned [46].

Our data illustrate how SMI may also contribute to sexual inactivity. Both men and women complained over decreased sexual desire, with one man explicitly stating that he was no longer capable of having sex. Participants attributed their sexual dysfunction to the mental illness and to the medication side-effects, similar to sexually inactive persons with SMI in the United States [18], and consistent with the notion that sexual dysfunction is common among persons with SMI [47]. Several men in our study also perceived the SMI as an obstacle to establishing stable intimate relationships, while only one woman mentioned this problem. In (urban) Uganda, a ‘masculine’ man is supposed to be the head of household and bread-winner for the family [48]. Inability to provide for a family due to SMI contradicts this masculine ideal, leading to low self-esteem and devaluating the man in the eyes of potential partners. In addition, chronic illness implies weakness which may further stigmatise men [49]. Indeed, failure to fulfil gender roles has been suggested to constitute a core component in the formation of mental illness stigma in other settings [17], [50]. Thus, a vicious circle could hypothetically operate where SMI and mental illness stigma contribute to men's sexual isolation, and where sexual isolation implies failed masculinity, in turn increasing stigma and further contributing to sexual isolation.

### Methodological considerations

Our findings are based on the accounts of persons with SMI and future studies should also include other perspectives, in particular those of partners and family members of persons with SMI.

Sexual behaviours and experiences are sensitive topics, and social desirability might have influenced what participants told us. However, research interviewers were Ugandan clinical psychologists experienced in communicating with persons with SMI, and took time establishing rapport before approaching sensitive topics. Moreover, participants did describe experiences that are not socially desirable in Uganda, such as having multiple partners (for women) or being sexually isolated (for men).

Some participants with schizophrenia showed passivity and had poverty of speech, resulting in interviewer-driven conversations with reduced richness of the data. In these cases, we were unable to develop a full understanding of the participants' lived experience.

We stopped the recruitment of men after seven participants, given that men's accounts were remarkably coherent. Although we felt that theoretical saturation was obtained, further interviews directly targeting men who are sexually active could potentially shed light on how SMI may impact on sexual health risks among sexually active men in Uganda.

Although we recruited study participants in contact with mental health services in the Ugandan capital, some aspects of our findings may be transferable also to persons with SMI living in rural Uganda. In fact, some of our findings (e.g. casual sex during illness episodes) may have particular relevance in rural Uganda, since access to psychiatric services is poor in rural areas.

### Implications

Our findings illustrate challenges to sexual health and rights among persons, especially women, with SMI in a Ugandan context. Some of these challenges could potentially increase the women's risk of HIV infection.

Targeted HIV interventions tailored to the specific needs of persons with SMI in Uganda are needed. Mental health services provide a logical venue for reaching persons with SMI, but interventions for patients in contact with mental health services would exclude the majority of persons with SMI in Uganda. Poor coverage of mental health services may not only increase sexual health risks, but also complicates HIV prevention. The need to prevent HIV transmission to and from persons with SMI provides an additional argument for scaling-up mental health services in resource-poor settings with high HIV prevalence.

Our study suggests that HIV interventions for persons with SMI need to be gender sensitive. The findings illustrate how the women with SMI in our study are not in control of their own sexuality, with sexual risk behaviours and sexual health risks to a large extent being shaped by their social environment. HIV interventions targeting women may need to include also families, partners and ultimately the societies where women with SMI live. For instance, education of family members about mental illness could increase women's social support and protect against sexual exploitation by partners. Information about the need for early help-seeking could prevent social instability and exposures to risky situations (e.g. homelessness) during illness episodes.

The neglect of the needs and human rights of persons with SMI is a human tragedy. The current study provides additional understanding of the difficult life situation of persons living with SMI in resource-poor settings, documenting stories of rejection by families, exploitation by partners, sexual abuse by health workers, and rape during illness episodes. Our findings suggest that a human rights approach may be needed for effective HIV prevention also for persons with SMI in sub-Saharan Africa, as it is for other marginalised groups at high risk of HIV.
